# 

**Published:** 1913-06

**Authors:** 


*The following donations have been received since the publication of the List in March.*

*May* 31*st*, 1913.

F. R. Cross (1) .. .. .. .. .. 3 volumes.
L. M. Griffiths (2) .. .. .. .. .. 12 ,,
E. W. Hey Groves (3) .. .. .. .. 1 volume.
A. H. Hooker (4) .. .. .. .. .. 1 ,,
R. Shingleton Smith, M.D. (5) .. .. .. 14 volumes.

Unbound periodicals have been presented by Mr. F. R. Cross and Mr. J. C. Heaven. Reprints have been received from Dr. James Swain.

### EIGHTY-EIGHTH LIST OF BOOKS.

The titles of books mentioned in previous lists are not repeated.

The figures in brackets refer to the figures after the names of the donors, and show by whom the volumes were presented. The books to which no such figures are attached have either been bought from the Library Fund or received through the *Journal*.

**Abel, R.** .. .. .. *Laboratory Handbook of Bacteriology* (Trans. from fifteenth German edition by M. H. Gordon) .. .. .. .. .. .. .. 2nd Ed. [1912]

**"Æsculapius Scalpel."** *Dying Scientifically* .. .. .. .. (2) 1888

**Alderson, W. E.** .. *Dental Anæsthetics* .. .. .. .. 2nd Ed. 1913

**Barker, W. R.** .. *The Bristol Museum and Art Gallery* .. .. 1906

**Batten and H. Thursfield, A. E. Garrod, F. E.** [Eds.]. *Diseases of Children* .. .. .. .. .. .. .. .. 1913

*Birmingham, Handbook of* (B.M.A.) .. .. .. .. .. .. .. .. 1911

**Bland-Sutton, Sir J.** *Fibroids of the Uterus* .. .. .. .. .. [1913]

**Brody, G. St.** .. *The Flora of Weston* .. .. .. .. (2) 1856

**Browne, Sir T.** .. *Religio Medici* (Ed. by W. P. Smith) .. (2) 1874

**Burghard, W. W. Cheyne and F. F.** *Manual of Surgical Treatment.* New Ed. (Ed. by T. P. Legg and A. Edmunds) .. .. .. .. .. .. Vol. IV 1913

**Burnet, E.** .. .. *The Campaign against Microbes* (Trans. by E. E. Austen) .. .. .. .. .. .. .. (5) 1909

**Cheyne and F. F. Burghard, W. W.** *Manual of Surgical Treatment.* New Ed. (Ed. by T. P. Legg and A. Edmunds) .. .. .. .. .. .. Vol. IV 1913

**Chundra, J. L.** .. *Laws of Sexual Philosophy* .. .. .. .. 1913

**Cotar, C.** .. .. *The Mineral Waters of Vichy* .. .. .. 1913

**Cornaro, L.** .. .. *Sure Methods of attaining a Long and Healthful Life* (Trans. by W. Jones) .. .. (2) 1768

| | | | |
|---|---|---|---|
| **Crooke, G. F.** | *Medical Pathology of Tuberculosis* | (5) | 1891 |
| **Culpeper, N.** | *The English Physician* (enlarged) | (2) | 1801 |
| **Dührssen, A.** | *Geburtschilfliches Vademekum* | 10th Ed. | 1913 |
| **Faust, B. C.** | *Catechism of Health* | (2) | [1832] |
| **Fernie, W. T.** | *Our Outsiders : and what they betoken* | | 1913 |
| **Flint, A.** | *Medicine of the Future* | (5) | 1886 |
| **Freud, S.** | *The Interpretation of Dreams* (Trans. by A. A. Brill) | | 1913 |
| **Gabbett, P. C.** | *Manual for Women's Voluntary Aid Detachments* | 2nd Ed. | [N.D.] |
| **Garrod, F. E. Batten and H. Thursfield, A. E.** [Eds.] | *Diseases of Children* | | 1913 |
| **Goodhart, Sir J. F.** | *The Passing of Morbid Anatomy* | (5) | 1912 |
| **Hanna, W.** | *Studies in Small-Pox and Vaccination* | | 1913 |
| **Hart, A. H.** | *How to Cut the Drug Bill* | 3rd Ed. | 1913 |
| **Hewer, Mrs. J. L.** | *Our Baby* | 14th Ed. | 1913 |
| **Hooker, A. H.** | *Chloride of Lime in Sanitation* | (4) | 1913 |
| **James, A.** | *Pulmonary Phthisis* | (5) | 1888 |
| **Jex-Blake, A. J.** | *Death by Etectric Currents and by Lightning* | | 1913 |
| **Johnson, J.** | *Excursions to the principal Mineral Waters of England* | (2) | 1843 |
| **Jones, H. L.** | *Medical Electricity* | 6th Ed. | 1913 |
| **Julliard, C.** | *Les Bandages* | 2nd Ed. | 1913 |
| **Langmead, F.** | *Forms of Mental Defect* | | 1913 |
| **Milne, R.** | *Home Treatment and Prevention of Scarlet Fever* | (5) | 1910 |
| **Molière, J. B. P. de** | *Le Médicin malgré lui* (Notes by G. E. Fasnacht) | (2) | 1895 |
| **Morland, C. Riviere and E.** | *Tuberculin Treatment* | 2nd Ed. | 1913 |
| **Murphy, J. K.** [Ed.] | *The Practitioner's Encyclopædia of Medicine and Surgery* | 2nd Ed. | 1913 |
| **Musgrove, C. D.** | *Nervous Breakdowns and How to Avoid Them* | | 1913 |
| **Parker, T. J.** | *Biographical Sketch of W. Kitchen Parker* | (5) | 1893 |
| **Pembrey and J. Ritchie, M. S.** [Eds.] | *General Pathology* | | 1913 |
| **Ritchie, M. S. Pembrey and J.** [Eds.] | *General Pathology* | | 1913 |
| **Riviere and E. Morland, C.** | *Tuberculin Treatment* | 2nd Ed. | 1913 |
| **Saundby, R.** | *Old Age* | | 1913 |
| **Sawyer, Sir J.** | *Insomnia* | 2nd Ed. | 1912 |
| **Sewill, H.** | *Dental Caries and Prevention* (5) | 2nd Ed. | 1888 |
| **Simon, A. L.** [Ed.] | *In Vino Veritas* | (5) | 1913 |
| **Thorne, L. T.** | *The "Nauheim" Treatment of Diseases of the Heart* | 4th Ed. | 1913 |
| **Thursfield, A. E. Garrod, F. E. Batten and H.** [Eds.] | *Diseases of Children* | | 1913 |
| **Tunstall, F. J. Warwick and A. C.** | *First Aid* | 8th Ed. | 1913 |
| **Walsh, D.** | *The Röntgen Rays in Medical Work* | (5) | 1897 |

**Warwick and A. C. Tunstall, F. J.** *First Aid* .. .. 8th Ed. 1913
**Williams, C. T.** .. *Old and New Views on the Treatment of Consumption* .. .. .. .. .. .. .. (5) 1911

### TRANSACTIONS, REPORTS, JOURNALS, &c.

American Association of Genito-Urinary Surgeons, Transactions of the.. .. .. .. .. .. .. .. .. .. Vol. VII 1912
American Journal of Ophthalmology, The .. .. Vol. XXIX 1912
American Journal of Surgery .. .. .. .. .. .. Vol. XXVI 1912
American Laryngological, Rhinological and Otological Society, Transactions of the .. .. .. .. .. .. .. .. .. 1912
American Medicine .. .. .. .. .. .. .. .. Vol. XVIII 1912
American Pediatric Society, Transactions of the .. Vol. XXIV 1912
Annales de Dermatologie et de Syphiligraphie .. Tom. III 1912
Annales d'Oculistique .. .. .. .. (1) Tom. CXIII, CXIV 1895
Archives of Pediatrics .. .. .. .. .. .. .. .. Vol. XXIX 1912
Boston Medical and Surgical Journal, The .. Vol. CLXVII 1912
British Journal of Dermatology, The .. .. .. Vol. XXIV 1912
British Journal of Tuberculosis, The .. .. .. Vols. V, VI 1911
Bulletin de l'Académie de Médecine .. Tom. LXVII, LXVIII 1912
Bulletin de la Société française de Dermatologie .. .. .. .. 1912
Canadian Medical Association Journal, The .. .. Vol. II 1912
Clifton College Register .. .. .. .. .. (2) 1880, 1887, 1890
Clinical Journal, The .. .. .. .. .. .. .. .. Vol. XL 1912
College of Physicians of Philadelphia, Transactions of the Vol. XXXIV 1912
Deutsche Zeitschrift für Chirurgie .. .. .. .. Bd. CXIX 1912
Dublin Journal of Medical Science, The .. .. (2) Vol. LXX 1880
Écho médical du Nord, L' .. .. .. .. .. .. .. .. .. .. .. 1912
English Catalogue of Books, The .. .. .. .. .. .. .. 1912
Gazette des Hopitaux .. .. .. .. .. .. .. .. .. .. .. .. 1912
Gazette des Hopitaux de Toulouse .. .. .. .. .. .. .. 1911
Hospitals—
  Johns Hopkins Hospital Reports, The .. .. Vol. XII 1904
  St. Bartholomew's Hospital Reports .. .. Vol. XLVIII 1913
Jahreskurse für Ärztliche Fortbildung .. .. .. .. .. (2) 1913
Johns Hopkins Hospital Bulletin, The .. .. Vol. XXIII 1912
Journal of Experimental Medicine, The .. .. .. Vol. XVI 1912
Journal of Laryngology, Rhinology and Otology, The Vol. XXVII 1912
Journal of Nervous and Mental Disease, The Vol. XXXIX 1912
Journal of Obstetrics and Gynæcology of the British Empire, The Vol. XXII 1912
Journal of the American Medical Association, The Vol. LIX 1912
Journal of the Royal Sanitary Institute .. .. Vol. XXXIII 1912
Journal of Tropical Medicine, The .. .. .. .. .. Vol. XV 1912
Library Association Record, The .. .. .. .. .. Vol. XIV 1912
Medical Annual, The .. .. .. .. .. .. .. .. .. .. .. 1913

| | | |
|---|---|---|
| Medical Record .. .. .. .. | Vols. LXXX, LXXXII | 1911–12 |
| Münchener medizinische Wochenschrift .. .. | Bd. II für | 1912 |
| National Association for the Prevention of Consumption, Transactions of the .. .. .. .. .. .. .. .. .. .. | (5) | 1911 |
| Post-Graduate, The .. .. .. .. .. .. .. | Vol. XXVII | 1912 |
| Practice of Medicine and Surgery by Unqualified Persons in the United Kingdom, Report as to the .. .. .. .. .. .. .. .. | | 1910 |
| Public Health .. .. .. .. .. .. .. .. .. | Vol. XXV | 1911–12 |
| Revue de Chirurgie .. .. .. .. .. .. .. .. .. | Tom. XLVI | 1912 |
| Revue de Thérapeutique médico-chirurgicale .. .. .. .. .. | | 1912 |
| Revue générale d'Ophtalmologie .. .. .. .. | Tom. XXXI | 1912 |
| Therapeutic Gazette, The .. .. .. .. .. .. | Vol. XXXVI | 1912 |

### MEETINGS OF SOCIETIES.

**University of Bristol.**—Students of the University have recently passed the following examinations :—

UNIVERSITY OF LONDON.—*Second Examination for Medical Degrees : Part I,* C. C. Chesterman. *Part II,* A. W. Adams ; (excluding pharmacology), F. K. Hayman.

M.B., CH.B. BRISTOL.—*Intermediate Examination :* C. N. Vaisey, Hilda K. Ewins, W. McKenzie, G. R. Wookey.

CONJOINT BOARD OF LONDON.—*Chemistry and Physics :* F. L. Fox, A. E. Young. *Biology :* W. A. Jackman. *Practical Pharmacy :* W. E. Neale. *Anatomy and Physiology :* H. R. W. Husbands, R. W. Little, D. Munro-Smith. *Medicine, Surgery and Midwifery :* W. Salisbury.* *Medicine :* H. W. Cooke, Miss Barnes. *Surgery :* Miss Barnes. *Midwifery :* A. G. Morris, R. B. Johnson, H. N. Morgan.

L.D.S. ENG.—*Mechanical Dentistry :* Marjorie J. White. E. W. Joyner. *Dental Metallurgy :* E. W. Joyner, W. H. Greet. *Final Examination (Part I) :* E. W. Thomas, J. D. Melhuish, *Part II :* K. Batten,* J. D. Melhuish.*

APPOINTMENTS.

**Bristol General Hospital.**—C. A. Morton, F.R.C.S., has been re-appointed Surgeon. E. W. Hey Groves, M.D., M.S. Lond., F.R.C.S., has been appointed Surgeon. A. J. Wright, M.B., B.S. Lond., F.R.C.S., Surgeon to the Nose and Throat Department ; and Clifford A. Moore, M.S. Lond., F.R.C.S., Assistant Surgeon ; J. Freeman, M.D., has been re-appointed Anæsthetist.

G. Hely-Hutchinson Almond, B.M., B.Ch. Oxon., has been appointed Honorary Pathologist to the Royal Mineral Water Hospital, Bath.

C. A. Joll, F.R.C.S., has been appointed Senior Resident Medical Officer, Royal Free Hospital.

A. Fells, M.B., C.M. Edin., has been appointed Honorary Surgeon to the Bristol Eye Dispensary.

* Qualified.

### *PUBLICATIONS RECEIVED.*

From FÉLIX ALCAN, Paris:
*Les Bandages, Pansements et Appareils Chirurgicaux.* Par le Dr. CHARLES JULLIARD. Deuxieme Edition. 1913. 10 fr.

From GEORGE ALLEN & COMPANY, LTD., London:
*The Interpretation of Dreams.* By Prof. Dr. SIGMUND FREUD, LL.D. Authorised translation of Third Edition by A. A. BRILL, Ph.B., M.D. 1913. 15s. net.

From EDWARD ARNOLD, London:
*Diseases of Children.* By Various Authors. Edited by ARCHIBALD E. GARROD, D.M., M.A. FREDERICK E. BATTEN, M.D., M.A., and HUGH THURSFIELD, D.M., M.A. 1913. 30s. net.
*Old Age.* By ROBERT SAUNDBY, M.D., 1913. 7s. 6d. net.
*Text-Book of General Pathology.* Edited by M. S. PEMBREY and J. RITCHIE. 1913 18s. net.

From J. W. ARROWSMITH LTD., Bristol:
*Nervous Breakdowns and How to Avoid Them.* By CHARLES D. MUSGROVE, M.D. 1913. 2s. 6d. net.

From BAILLIÈRE, TINDALL AND COX, London:
*The "Nauheim" Treatment of Diseases of the Heart and Circulation.* By LESLIE THORNE THORNE, M.D., B.S. Fourth Edition. 1913. 3s. 6d. net.

From JOHN BALE, SONS & DANIELSSON, LTD., London:
*The Practitioner's Vade Mecum, or How to Cut the Drug Bill.* By A. HERBERT HART, M.S., M.D. Third or National Insurance Edition. 1913. 2s. 6d. net.

From J. L. CHUNDRA, Calcutta:
*Laws of Sexual Philosophy: an exposition of Eastern and Western Sexual Science from Medical, Moral and Social Point of View.* By J. L. CHUNDRA, L.M.S. 1913. 4s. net.

From J. & A. CHURCHILL, London:
*Forms of Mental Defect.* By FREDERICK LANGMEAD, M.D. 1913. 1s. net.

From CORNISH BROS., Birmingham:
*Insomnia: its Causes and Treatment.* By Sir JAMES SAWYER. Second Edition. 1912

From HENRY FROWDE AND HODDER AND STOUGHTON, London:
*Abel's Laboratory Handbook of Bacteriology.* Second English Edition. Translated from the Fifteenth German Edition by M. H. GORDON, M.A., M.D. [1912.] 5s. net.
*Tuberculin Treatment.* By CLIVE RIVIERE, M.D., and EGBERT MORLAND, M.B. Second Edition. 1913. 6s. net.
*The Practitioner's Encyclopædia of Medicine and Surgery.* Edited by J. KEOGH MURPHY, M.D., F.R.C.S. Second Edition. 1913. 35s. net.

From Dr. A. J. JEX-BLAKE, London:
*Death by Electric Currents and by Lightning.* The Goulstonian Lectures for 1913. By A. J. JEX-BLAKE, M.A., M.B., B.Ch. 1913. [Reprint.]

From S. KARGER, Berlin:
*Geburtschilfliches Vademekum.* Von Prof. Dr. A. DÜHRSSEN. Zehnte Auflage. 1913. M. 5.60.

From H. K. LEWIS, London:
*The Mineral Waters of Vichy.* By CHARLES COTAR, M.D. With a Foreword by VAUGHAN HARLEY, M.D. 1913. 4s. net.
*Medical Electricity.* By H. LEWIS JONES, M.A., M.D. Sixth Edition. 1913. 12s. 6d. net.

From LONGMANS, GREEN AND CO., London:
*A Manual of Surgical Treatment.* New Edition. Entirely revised with the assistance of T. P. LEGG, M.S., F.R.C.S., and ARTHUR EDMUNDS, M.S., F.R.C.S. Vol. IV. 1913. 21s. net.

From SCIENCE REVIEWS, LIMITED, London:
*Fibroids of the Uterus: their Pathology, Diagnosis and Treatment.* By Sir JOHN BLAND-SUTTON. [1913.] 4s. 6d. net.

From JOHN WRIGHT AND SONS LTD., Bristol:
*Studies in Small-Pox and Vaccination.* By WILLIAM HANNA, M.A., M.D., D.P.H. 1913. 7s. 6d. net.
*The Medical Annual.* 1913. 8s. 6d. net.
*Dental Anæsthetics.* By WILFRED E ALDERSON, M.D. Second Edition. 1913. 3s. net.
*Invalid and Convalescent Cookery.* By MARY E. BIRT. Second Edition. 1913. 6d. net.
*Manual for Women's Voluntary Aid Detachments.* By P. C. GABBETT, M.R.C.S. Second Edition. [N.D.] 1s. net.
*"First Aid" to the Injured and Sick.* By F. J. WARWICK, B.A., M.B., and A. C. TUNSTALL, M.D., C.M. Eighth Edition. 1913. 1s. net.
*Our Baby: for Mothers and Nurses.* By Mrs. J. LANGTON HEWER. Fourteenth Edition. 1913. 1s. 6d. net.
*Our Outsiders: and what they betoken.* A Summary. By W. T. FERNIE, M.D. 1913. 4s. 6d.

